# Population Level Inference for Multivariate MEG Analysis

**DOI:** 10.1371/journal.pone.0071305

**Published:** 2013-08-05

**Authors:** Anna Jafarpour, Gareth Barnes, Lluis Fuentemilla, Emrah Duzel, Will D. Penny

**Affiliations:** 1 Institute of Cognitive Neuroscience, University College London, London, United Kingdom; 2 Institute of Cognitive Neurology and Dementia Research, Otto-von-Guericke University, Magdeburg, Germany; 3 Wellcome Trust Centre for Neuroimaging, University College London, London, United Kingdom; 4 Cognition and Brain Plasticity Unit, Institute of Biomedicine Research of Bellvitge, Barcelona, Spain; 5 Department of Basic Psychology, University of Barcelona, Barcelona, Spain; University G. D'Annunzio, Italy

## Abstract

Multivariate analysis is a very general and powerful technique for analysing Magnetoencephalography (MEG) data. An outstanding problem however is how to make inferences that are consistent over a group of subjects as to whether there are condition-specific differences in data features, and what are those features that maximise these differences. Here we propose a solution based on Canonical Variates Analysis (CVA) model scoring at the subject level and random effects Bayesian model selection at the group level. We apply this approach to beamformer reconstructed MEG data in source space. CVA estimates those multivariate patterns of activation that correlate most highly with the experimental design; the order of a CVA model is then determined by the number of significant canonical vectors. Random effects Bayesian model comparison then provides machinery for inferring the optimal order over the group of subjects. Absence of a multivariate dependence is indicated by the null model being the most likely. This approach can also be applied to CVA models with a fixed number of canonical vectors but supplied with different feature sets. We illustrate the method by identifying feature sets based on variable-dimension MEG power spectra in the primary visual cortex and fusiform gyrus that are maximally discriminative of data epochs before versus after visual stimulation.

## Introduction

Multivariate analysis of Magnetoencephalography (MEG) data is a powerful technique which considers the relationship between multiple data features and multiple experimental conditions. Previously this approach has been used to study oscillatory representations of stimuli; such as visual stimuli at the time of perception [1], [2] or the replay of oscillatory patterns during memory tasks [3], [4]. A major problem with such multivariate analyses is the identification of discriminative data features from a high dimensional measurement space. In the above studies, the set of discriminatory features were allowed to vary from subject to subject. This between-subject variability, however, makes it difficult to interpret experimental findings in terms of a consistent set of underlying cognitive processes.

In this paper we present a principled approach to systematically select the most discriminatory and minimally complex feature set that is consistent over subjects ie. at the ‘group level’. This analysis also enables systematic inference on the dimensions of these feature-spaces. For example, if there is no dependence between data features and experimental condition the inferred dimension will be zero.

Our framework is based on Canonical Variates Analysis (CVA). CVA models multivariate dependencies between a set of class labels and data features. The order of the CVA model is then based on the number of significant canonical vectors, as determined by the Bayesian Information Criterion (BIC) [5], [6]. Absence of a multivariate dependence (or no significant decodability) is indicated by the zeroth order model (null model or model 0) being the most likely. Here we apply CVA to beamformer reconstructed MEG data in source space [7]. Nevertheless, in principle, it could be applied to data in sensor space or to data after various transformations, including the use of principal or independent component analysis [8]. (We return to this latter issue in the discussion.)

The model ranking approach allows us to test, at the group level, both whether there is a multivariate dependence between data features and experimental condition, and if there is, to find which feature sets maximise the strength of this dependence.

To test whether these multivariate dependencies are consistent over a group of subjects, we use random effects Bayesian model selection [9], based on the BIC values. We illustrate this method to determine the spectral resolution (number of frequency bands) that maximizes decodability of data features into the experimental conditions. We used MEG power spectra at each voxel in source space, within the regions of interest (ROIs), the primary visual cortex (V1) and fusiform gyrus (FFG), and the experimental conditions indicating whether the data were from a pre- or post-stimulus epoch of a simple visual processing paradigm.

## Materials and Methods

### Methods

This section describes the data processing pipeline we propose. This comprises five steps

1. **MEG Source Reconstruction**, This step is optional, as MEG data can also be analysed in sensor space or, for example, projected onto principal or independent component spaces. In this paper the features of the MEG signal we use are power spectra. More generally, these can be any function of the MEG data, such as phase and/or amplitude or more exotic nonlinear measures.
2. **Canonical Variates Analysis**, Here we apply a CVA model at each point in source space as our goal is brain mapping. The maps indicate which areas show consistent relationships between multivariate data features and experimental condition.
3. **Bayes factors**, The order of a CVA model is determined by the number of canonical vectors. This step computes the evidence of a model with 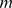 canonical vectors in relation to the evidence of a model with zero canonical vectors. The ratio of these evidences is known as a Bayes factor.
4. **Feature Set Selection**, The optimal model will depend not only on the number of canonical vectors but also on the features to which these vectors map. In this paper we compare models with single canonical vectors but with a different fractionation of the MEG power spectrum.
5. **Random Effects Bayesian Model Selection (RFX-BMS)**, The previous steps are applied to data from multiple subjects to produce Bayes factor maps for each subject and model comparison. This allows for single alternative models to be compared with a null model, or for any number of models to be simultaneously compared with each other. This final step computes the frequency with which models are used in the population from which the subjects are drawn.

The following subsections describe each of the above steps in more detail.

#### MEG Source Reconstruction

We source reconstructed data for each subject using the SPM8 implementation of the Linearly Constrained Minimum Variance (LCMV) beamformer [10]–[12]. The software for source reconstruction, and computation of Bayes factors for CVA models is available in the SPM Beamforming toolbox (http://code.google.com/p/spm-beamforming-toolbox/). This produces a log Bayes factor image for each subject and model. The software for implementing Random effects Bayesian model selection is available in the latest release of SPM [13] (http://www.fil.ion.ucl.ac.uk/spm/software/). This takes the log Bayes factor images for all subjects and produces expected frequency maps (*_xpm.img) and, optionally, exceedance probability maps (*_epm.img).

The forward model used in source reconstruction was defined using an inverse normalized canonical head-shape brain for all subjects [14]. At each source location we selected the orientation that maximises projected power [12] which gives a single weight vector for each source location. Briefly, the weights for location 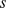 were given by
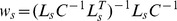
(1)where 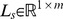 is the lead field matrix for 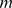 channels at source location 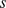 and 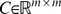 is the sensor covariance matrix. This corresponds to an LCMV beamformer with zero for the regularisation parameter [11]. Accordingly, the source level estimate of activity for trial 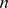 at location 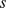 is given by
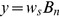
(2)where 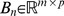 comprises 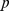 complex valued Fourier coefficients describing the signal at 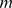 MEG sensors on trial 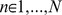. In the next section we go on to look at multivariate dependence between the experimental design and the spectral features and this source level estimate across the brain.

#### Canonical Variates Analysis

CVA is a method for detecting dependencies between a set of variables 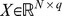, and a set of variables 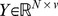. The aim of CVA is to find the linear projections of 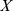 and 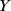 with maximal correlation. Given 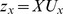 and 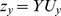, we can compute the canonical correlation
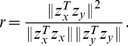
(3)The projections 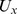 and 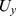 which maximise this correlation are known as the canonical vectors and the resulting 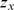 and 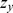 are the canonical variates. If 
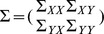
 is the sample covariance matrix and 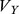 and 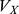 are the left and right singular vectors of 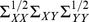 in decreasing order, then the canonical vectors can be computed as 
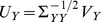
 and 
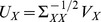
 [15]. There are 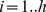 pairs of canonical vectors where 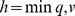. The canonical correlations 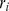 for 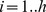 are used to compute Bayes factors, as described in the following section.

#### Bayes Factors

We first introduce some terminology. The dimension of a CVA model is given by the number of significantly non-zero canonical vectors. If there exists a linear multivariate dependence between 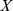 and 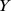 then the dimension of the corresponding CVA model is non-zero. Thus one can test for linear multivariate dependence by estimating CVA model dimension.

A standard approach from classical inference here is Bartlett's test for dimensionality [15]. However, to our knowledge, there is no simple way to carry over these results to the group level. We therefore prefer a Bayesian method, as this integrates seamlessly with established methods for group level inference (see final subsection).

These Bayesian methods first compute the evidence for a model, 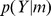, with 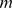 canonical vectors. Various methods exist for computing the Bayesian model evidence for a CVA model. These include the Bayesian Information Criterion (BIC) [5], [6] and variational approximations [16]. This paper uses a BIC approximation which we now derive.

If there is no relation between dependent variable (or ‘data’) 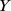 and independent variable 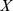, then the log-likelihood of the data is
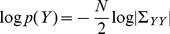
(4)where 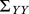 is the data covariance. If there is a relation between 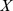 and 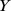 then the log-likelihood can be calculated as follows. The maximum likelihood coefficients are 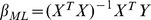 and the log-likelihood is
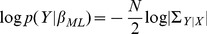
(5)where 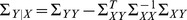, 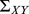 is the covariance between 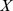 and 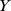, and 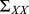 is the covariance of 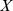. The log-likelihood ratio, 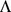, is therefore
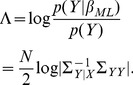
(6)If 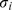 is the 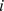
th eigenvalue of 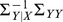 we can write
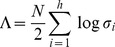
(7)where 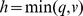. This is also known as Wilk's Lambda [15]. We also define the quantity
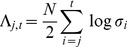
(8)so 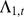 is the log-likelihood ratio for a CVA model with 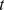 canonical variates. The quantity 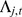 is used to compute the BIC and can also be expressed in other forms. We next show how it is computed in our implementation, and finally show how it is related to canonical correlations.

A second expression for 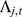 can be derived as follows. Let 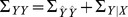, where 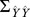 is the covariance explained by the model and 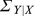 is the covariance not explained by the model. Then if 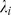 are eigenvalues of 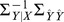 then the above relationship can be used to show that 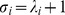 (see Appendix B of [17]). Hence an alternative expression for Wilk's Lambda is
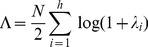
(9)This expression is used in the current paper and has been implemented in the SPM software [17]. Accordingly, 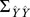 can be formed directly from model predictions
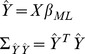
(10)and 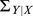 from the residuals
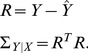
(11)


The 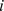
th canonical correlation can be expressed as 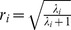. Hence, a third equivalent form for the log likelihood ratio is
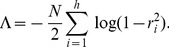
(12)In summary, we can write
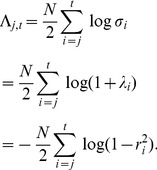
(13)This last expression appears in [5], [6].

The log evidence for a model with no parameters (null model) is simply the log likelihood of the data, 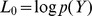. The log evidence for model 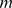 with parameters 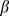 is given by 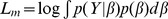. This can be approximated by the Bayesian Information Criterion (BIC) as
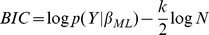
(14)where 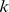 is the number of parameters in the model and 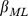 are the maximum likelihood parameters. A Bayes factor is the ratio of model evidences. Here we define
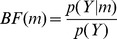
(15)Log Bayes factors can therefore be approximated as differences in the BIC scores. Under BIC, the log Bayes factor for a CVA model of dimension 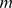 versus a model with dimension zero (null model) is given as
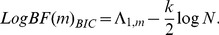
(16)where
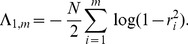
(17)and 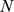 is the number of data points and 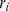 are the canonical correlations at each dimension 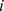. This expression has been used in previous studies [5], [6] and is derived from equation 13. The estimated model order is the one which has the largest LogBF. Negative values of 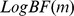 express evidence in favour of the null model. Intuitively, better CVA models will have stronger canonical correlations (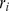) and fewer parameters (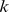).

#### Feature Set Selection

It is also possible to compute Bayes factors for models with the same number of canonical vectors but supplied with different feature sets. Bayesian model comparison here allows the models to vary but the data must stay the same. Feature set selection therefore requires that we set up CVA models such that 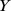 is a design matrix encoding experimental conditions and 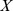 are independent variables comprising the neuroimaging data features (in other words we switch the traditional roles of these variables (X and Y) to make it clear that we are searching for optimal data features for a fixed experimental design). In this paper these features are 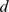-dimensional power spectra. We then compute 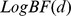 images where each is the log Bayes Factor for a model with a single canonical vector and 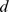-dimensional features 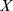, versus a model with zero canonical vectors. We can then use the same Bayes factor images to compare different feature dimensions. For example, for pairwise comparisons
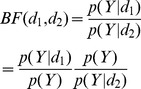
(18)Hence
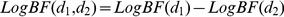
(19)We can also implement multi-way comparisons as described in the next section.

#### Random effects Bayesian model selection

Random effects Bayesian model selection (RFX-BMS) [9] views the assignment of models to subjects as a random process in which each subject is assigned to model 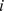 with probability 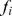. Here 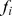 is the frequency with which model 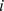 is used in the population from which the subjects were drawn. The Bayesian algorithm for estimating model frequencies 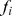 from the table of log model evidence values [9] uses a Dirichlet prior
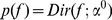
(20)with ‘count parameters’ 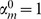. These parameters can be thought of as corresponding to the assumption of having previously observed one instance of each model type. These parameters produce a flat prior. The posterior is approximated to also be a Dirichlet
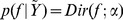
(21)where 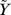 indicates data from all subjects. The count parameters 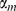, are initialised as 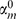, and then updated iteratively as follows
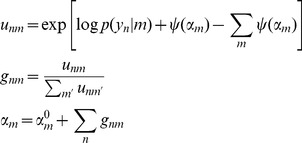
(22)where 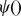 is the digamma function [18]. Here 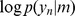 is the entry in the log evidence table from the 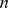
th subject (row) and 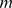
th model (column). The quantity 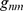 is the posterior probability that subject 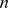 used the 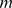
th model. This is ‘posterior’ as in after seeing the model evidence table and, implicitly, the data from all subjects 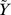.

This algorithm can also be applied to a table of log Bayes factor values, as long as the Bayes factors have all been computed with respect to the same common model. In this paper, the common model is the null CVA model with zero canonical variates.

The goal of RFX-BMS is to estimate 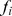 using a table of model evidence scores, or Bayes factors with respect to a common model, from 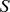 subjects and 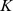 models. Intuitively, if the scores favour model 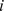 in 9 out of 10 subjects, then 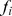 will be estimated to be about 0.9. However, the estimate of 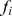 is also influenced by the degree to which models are favoured. For example, if for the 10th subject the score is greatly in favour of a different model then the estimate of 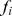 will be commensurately reduced. Given data 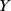 (in practice, a table of logBF values), a posterior distribution, 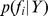, can be estimated using the algorithm described in [9]. The mean of this distribution, 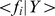 provides an estimate of the model frequencies. This is also referred to as the ‘expected frequency’.

**Figure 1:**
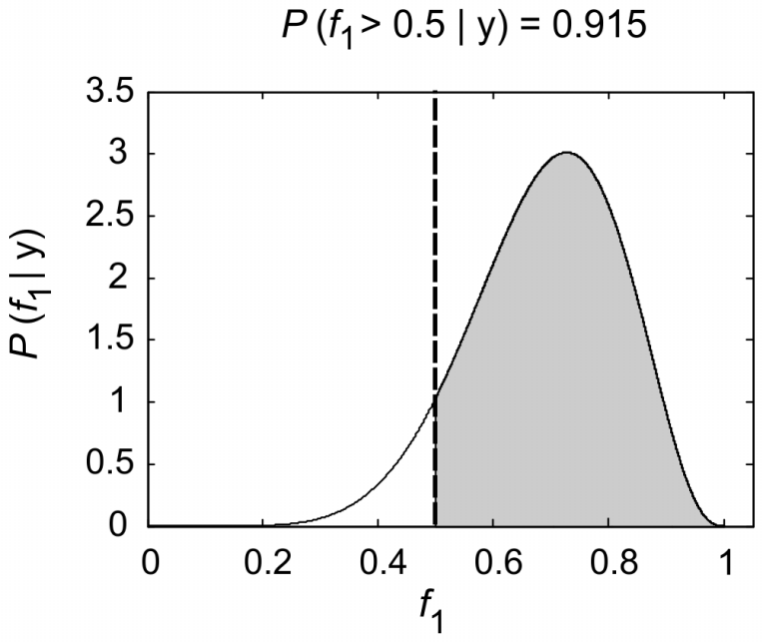
This graphic demonstrates random effects model selection for the case of comparing two models. These models have frequencies 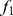 and 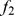. These frequencies refer to the population from which the subjects were drawn. The figure plots the posterior probability of 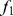. The mean of this density is 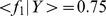, indicating that 75% subjects use model 1. For two models, the exceedance probability 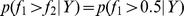 is given by the posterior mass in excess of 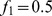. Here, the exceedence probability is 0.915.

It is also possible to compute the probability that one model frequency exceeds another. For example, when comparing just two models we can compute 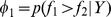. This is known as the exceedance probability for model 1 over model 2. Figure 1 illustrates the concept of an exceedance probability. If one has maps of model evidence over anatomical space, and for multiple subjects, it is possible to produce maps of expected frequencies or exceedance probabilities. In previous work [19], for example, Exceedance Probability Maps (EPMs) were plotted for univariate General Linear Models fitted to functional MRI data. In this paper we plot expected frequency maps for CVA models in MEG source space.

### Experimental Data

#### Participants and Experiment

10 healthy young adults (6 female; on average 23 years old (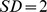)) participated in an episodic memory study. All participants gave written informed consent to participate and the study was approved by the University College London Research Ethics Committee for human-based research. All participants were financially compensated for their participation. The overall goal of this study was to examine the neural correlates of visual memory. Here, we used the data from a small part of this study in which subjects were presented with images of faces. These images were grey scaled and normalized to a mean grey value of 127 and SD of 75, of dimensions 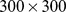 pixels, and shown upon a grey background (grey value of 127) subtending approximately 6 degrees of horizontal and vertical visual angle.

#### MEG recordings

MEG data were recorded with a 274 channel CTF Omega whole-head gradiometer system (VSM MedTech, Coquitlam, BC, Canada) with a 600 Hz sampling rate. Head position inside the system was acquired via head localizer coils attached to the nasion and 1 cm anterior to the left and right pre-auricular points. Participants were seated upright and the stimuli were back-projected onto a screen approximately 1 m in front of them.

### Data Analysis

At each point in source space we generated a 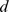-dimensional feature vector of power in 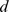 frequency bands. Here we used 6 different features. The frequency bands are as defined in Table 1. The average power in the frequency band was computed separately across 1 second before (−1000 to 0) and 1 second after (0 to 1000 ms) onset of visual stimulus epochs.

**Table 1:** Definition of feature space.

d	Frequencies(Hz)
1	3–90
3	3–10, 10–30, 30–90
5	3–8, 8–12, 12–30, 30–50, 50–90
7	3–5, 5–8, 8–12, 12–20, 20–30, 30–50, 50–90
9	3–5, 5–8, 8–10, 10–12, 12–20, 20–30, 30–40, 40–50, 50–90
11	3–5, 5–8, 8–10, 10–12, 12–20, 20–30, 30–40, 40–50, 50–60, 60–70, 70–90

This describes the fractionation of the power spectrum into 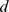 separate bands, 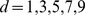 and 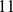.

**Figure 2:**
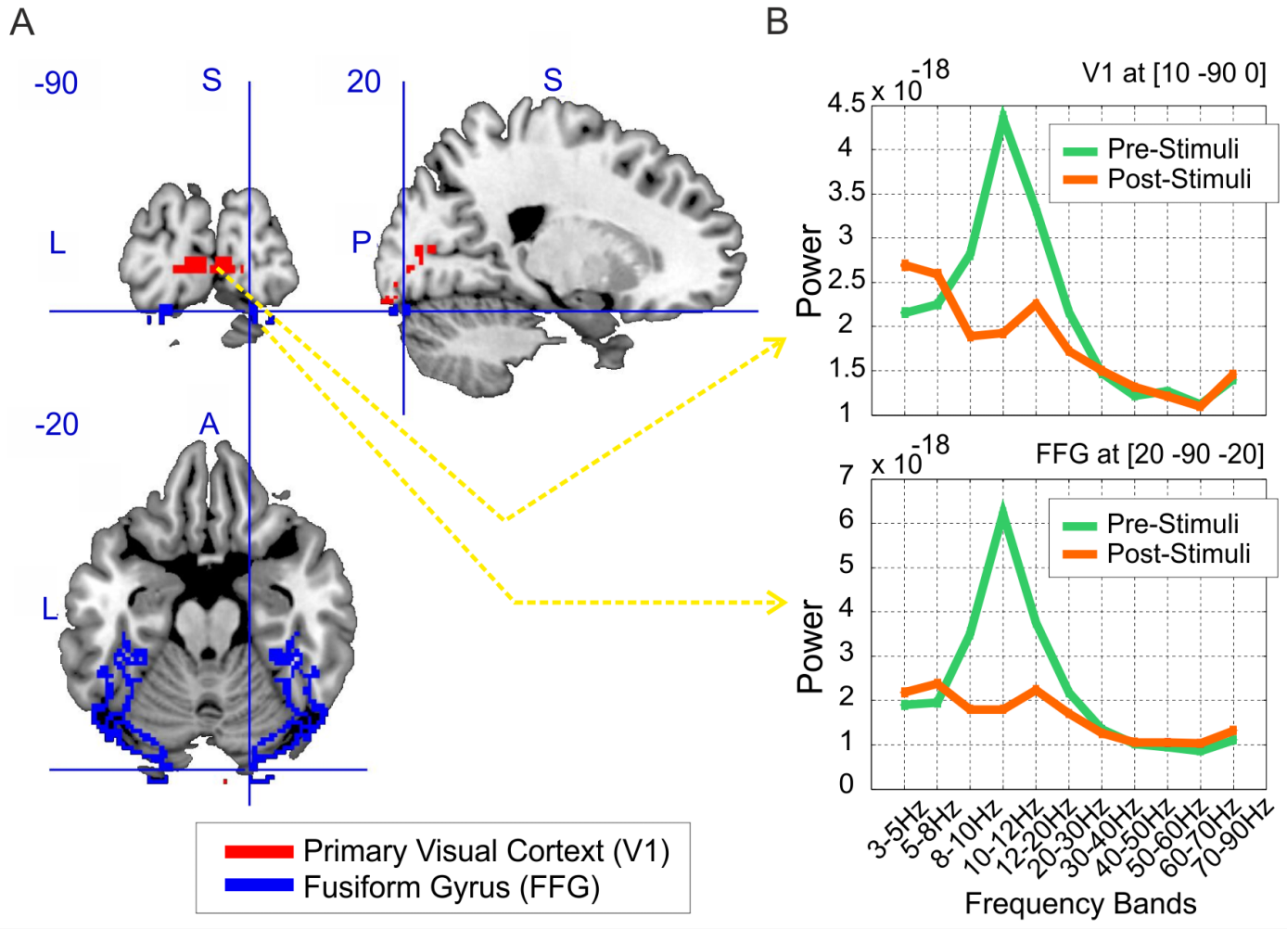
Regional Activity. (A) the MNI mask for region of interest: grey matter in the primary visual cortex (V1) and fusiform gyrus (FFG), the view is from [20 −90 −20]mm. (B) The power spectrum for pre- and post- stimulus activity across 11 frequency bands for an individual participant. The top plot is the signal from a V1 voxel, [10 −90 0]mm (Talairach coordinates), and the bottom plot is the signal from a FFG voxel, [20 −90 −20]mm.

We analysed data in two regions of interest, V1 and FFG, defined using the MNI grey matter masks shown in Figure 2A. We excluded any voxels which overlapped in the low-resolution source localization grid space (10 millimetres resolution). The FFG mask included 1600 voxels and the V1 mask included 574 voxels.

In this paper, our 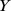 variable contains class labels with a scalar 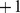 indicating post-stimulus, and a 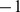 indicating pre-stimulus. We have 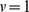. Therefore our CVA model has at most a single canonical component, 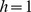. Our 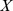 variable contains the 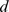-dimensional power spectra. Thus, the number of parameters in the CVA model is 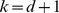.

The matrix 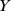 was prepared in the following way. Let each 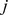 index Fourier bins within one of the pre-defined spectral bands ranging from 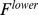 to 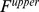 frequency. Then at each source location 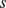 the activity at trial 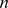 is formulated as 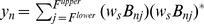 based on equation 2, where 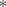 signifies the complex conjugate. In order to give equal weighting to all frequency bands (some of which have markedly less power) for each band we removed the mean value (across all conditions) and normalized the variance (in power) to unity.

For each subject we computed the 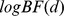 maps for 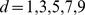 and 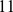. Each 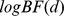 map is the log-evidence for a model with a single canonical vector, and 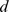 signal features, minus the log-evidence of the null model (no canonical vectors).

## Results

We studied 3–90 Hz oscillatory activity in pre- and post- visual stimulus presentation (an unfamiliar face) in source space, specifically in the primary visual cortex (V1) and fusiform gyrus (FFG). The aim of our analysis was to decode the differences in pre versus post stimulus activity, based on different feature sets (see Table 1 in the Methods and Material section). We hypothesised that there will be differences elicited by onset of the stimuli in the regions of interest. These regions were defined using the anatomical masks based on MNI brain (Figure 2A). In the first step, the MEG data for each individual participant was source localized and the features were defined as average power within the specified frequency bands. Figure 2B illustrates the average power across the 11 frequency bands (Model 11) in selected voxels from V1 and FFG. In the next step we studied which feature space best decoded the signals in each ROI based on the experimental design.

Firstly, we separately compared each of the models 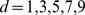 and 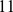 to the null model. Table 2 summarizes the results of these pairwise comparisons for each ROI. We compute the percentage of voxels with posterior expected frequencies greater than 0.9. For V1, the clearest result is that model 3 is better than the null in 88.8% voxels. For FFG, the findings are less clear cut, with both models 3 and 5 being better than the null in 38.4% and 27.3% voxels, respectively. These findings imply that the null model is better than the alternative models at a large proportion of voxels in FFG. We investigated this further with a set of multi-way model comparisons.

**Table 2:** Pairwise model comparisons versus the null.

ROI	Model 1	Model 3	Model 5	Model 7	Model 9	Model 11
FFG (1600 voxels)	64 (4.0%)	615 (38.4%)	438 (27.3%)	239 (14.9%)	148 (9.2%)	103 (6.4%)
V1 (574 voxels)	32 (5.5%)	510 (88.8%)	466 (81.1%)	362 (63.0%)	205 (35.7%)	58 (10.1%)

Total (and percentage) of voxels with posterior expected frequencies 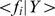 greater than 0.9. These are voxels where higher (than zeroth) order models are favoured in more than 90% of the population.

**Figure 3:**
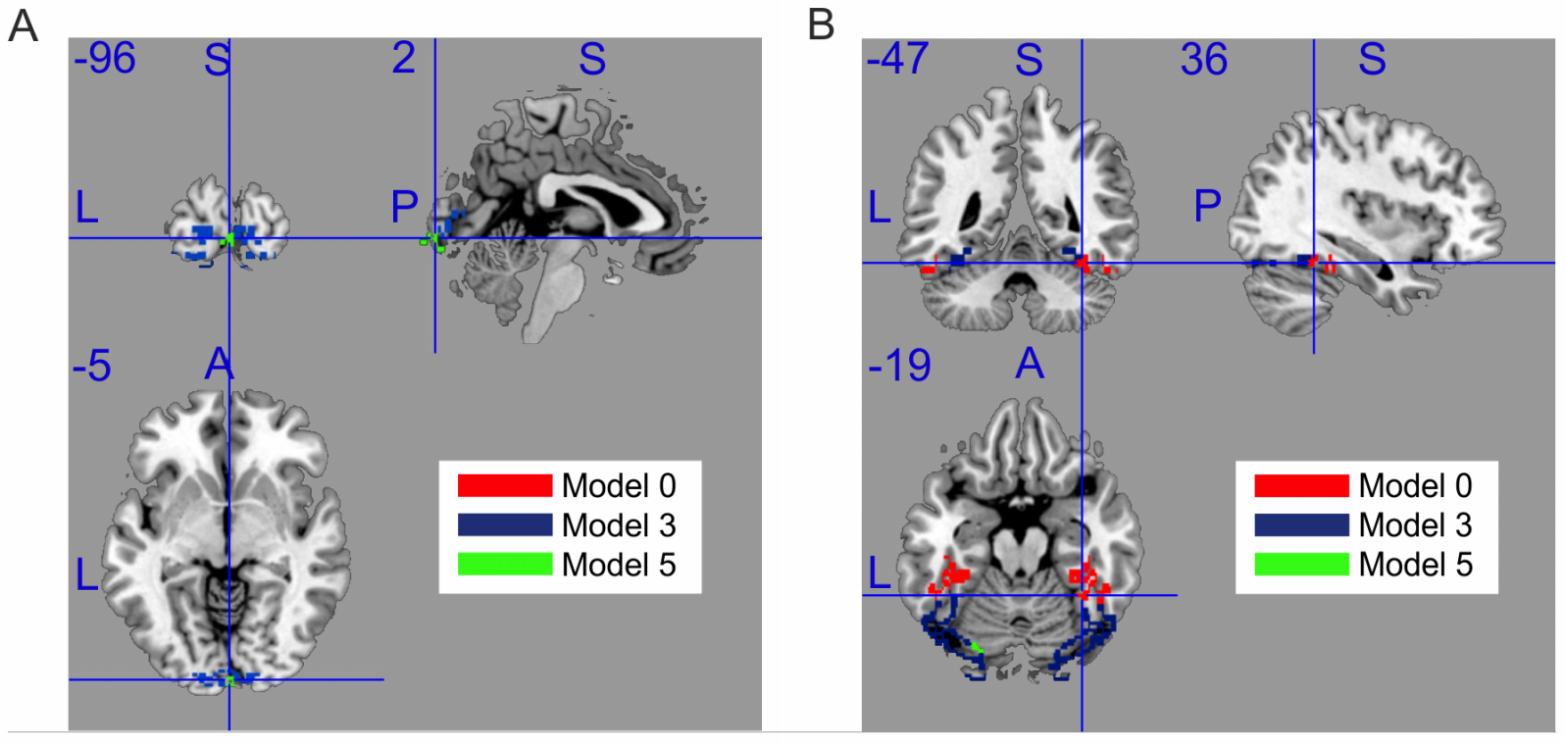
Multi-way model comparison maps. The maps show voxels where pre- versus post-stimulus activity is best discriminated by each model, for V1 (left) and FFG (right). (A) V1 view from [2 −96 −5]mm (B) FFG view from [36 −47 −19]mm (Talairach coordinates). Models 1, 7, 9 and 11 were not best at any voxel. Note that the null model is best for anterior FFG, but model 3 is best for posterior FFG.

For the multi-way comparisons we first created a model-comparison map with voxels colored to show which model has the highest frequency 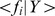 in the population. Figure 3 shows the results. Note that models 1, 7, 9 and 11 do not have the highest frequency at any voxel. The figure shows that model 3 is favoured in posterior FFG whereas the null model is favoured in anterior FFG. We can therefore infer that, in FFG, stimulus-induced changes in power spectra are restricted to posterior regions.

We also report summary statistics collapsed across all voxels in each region. Table 3, for example, shows the percentage of voxels in which the different models are favoured. For FFG, the null model is favoured in half of the voxels and, as we have seen in Figure 3, these voxels are in the anterior region. For V1 model 3 is favoured in 90.8% voxels.

**Table 3:** Multi-way between model comparison.

ROI	Model 0	Model 1	Model 3	Model 5	Model 7	Model 9	Model 11
FFG (1600 voxels)	812 (50.7%)	0	769 (48.1%)	19 (1.2%)	0	0	0
V1 (574 voxels)	1 (0.1%)	0	521 (90.8%)	52 (9.1%)	0	0	0

Total (and percentage) of voxels where model 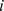 is the most favoured model (ie. has the largest posterior expected frequency 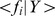).

A subtlety with random effects model selection is that the expected frequencies depend on the models in the comparison set. For example, in a pairwise (two-way) comparison model 3 beats model 0 at 510 voxels (see Table 2). Whereas in a multi-way comparison model 3 wins at 521 voxels (Table 3). The 11 extra voxels reflect spectral differences that were previously attributed to model 0, but given a wider comparison set are attributed to model 5. This is analogous to voting in elections where the addition of an extra option can ‘split the vote’ [20].

**Figure 4:**
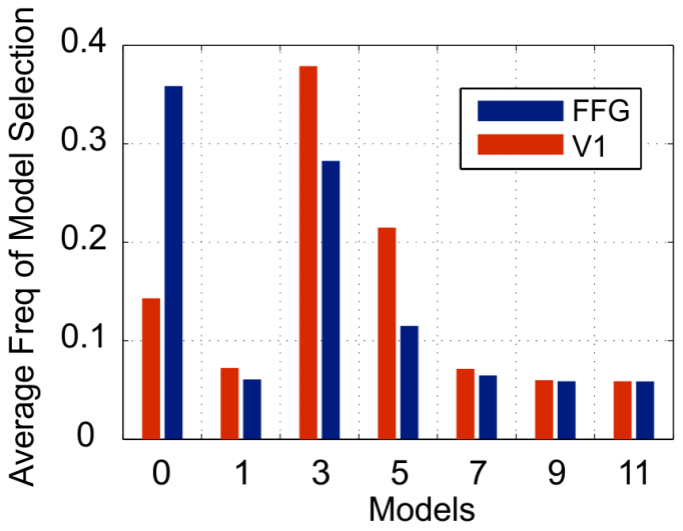
Average frequency of model selection. For each model we average the posterior expected frequencies 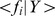 across all voxels in each ROI (FFG in blue and V1 in red). Models 3 and 5 are, on average, the most frequently selected in V1, and models 0 and 3 are the most frequently selected in FFG.

Finally, we report expected model frequencies averaged over all voxels in each ROI. This is shown in Figure 4. Models 3 and 5 are, on average, the most frequently selected in V1, and models 0 and 3 are the most frequently selected in FFG.

## Discussion

Here we proposed a solution to the problem of group-level inference from multivariate modelling of MEG data. The combination of scoring CVA models using BIC and assessing consistency across a group using Bayesian random effects model inference provides a principled solution. We applied this approach to source space power spectra in regions of interest to decode pre- versus post-stimulus epochs, using various feature sets (models). These feature sets differed in the degree to which the power spectra were fractionated (number of frequency bands). We were able to show that although all features sets provided some degree of discrimination between experimental conditions; the optimal feature set in general consisted of three approximately classical bands (3–10, 10–30, 30–90 Hz). Although our features sets were selected simply to illustrate the new approach, it is clear that the multivariate models outperformed univariate models even for this simple decoding task.

An optimal statistical model provides a balance between model fit and model complexity [21] and this general principle naturally applies to the CVA models we have employed. In this paper model complexity is assessed by the number of model parameters (see equation 16 where 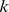 is the number of parameters). Thus, the univariate model (model 1) has low complexity (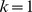) but is suboptimal at most voxels because it has a poor model fit (it has a low correlation with the experimental design). Conversely, model 11 has a good model fit but is suboptimal because it has a large complexity (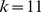); the improvement in the canonical correlation is not justified by the larger number of parameters.

Our data analyses focussed on inferring the optimal feature dimension across various exemplar feature sets. But there are also other ways in which random effects Bayesian model selection can be used for finding the optimal feature set. These include, for instance, fixing the number of features, but changing the feature set (eg. by breaking up spectra in a different way, or using phase/amplitude or more exotic nonlinear features). In our study the multivariate features were power spectra at single source voxels in regions of interest. But one could also apply the approach to data from local regions of voxels as in ‘searchlight’ approaches [22].

A perhaps subtle aspect of the RFX-BMS approach is that it is concerned only as to whether, for example, more subjects use model A than model B. This does not require that the parameters of the winning model are consistent over that group. For example, our model comparisons generally showed multiple voxels in which post-stimulus activity was better discriminated from pre-stimulus activity when the spectrum was described using a triplet (power in low, medium and high frequencies) rather than a scalar (power across all frequencies). This does not necessarily mean that the pattern of frequency responses was consistent over subjects. For example, half the subjects may have increases in low frequency power post-stimulus, and the other half decreases. One way to directly test for this scenario using the same scheme would be to see if a model using a fixed canonical vector over subjects (essentially a univariate test with data projected onto a single canonical vector) has more evidence than a model in which the canonical vector is allowed to vary (as here). Similar model comparison approaches could be used to test for differences (e.g. in the feature set) between different study groups, such as a patient group and a control group.

A large amount of neuroimaging research implements multivariate analysis using pattern recognition approaches based on artificial neural networks or support vector machines. A further benefit of the approach described in this paper is that model optimality is assessed using Bayes Factors, whereas the optimality of most pattern recognition approaches is assessed using cross validation [23]. Our approach is therefore more computationally efficient. For example, in the case that the assessment of model optimality is based on 10 fold cross-validation, our approach is 10 times faster.

We have applied our approach to beamformer reconstructed MEG data in source space. As noted in the introduction, it could also be applied to data in sensor space or data projected onto independent components [8]. As one of our goals has been to find data features that are consistent across a group of subjects, it would therefore also be necessary to use independent components that are consistent across the group. Fortunately, there are already established methods for doing this based on group-wise significance testing [24] or clustering [25].

This paper was based on MEG data; however, the combination of scoring CVA models and random effects model inference is not limited to MEG data and can be applied to any neuroimaging modality. In fMRI, for example, CVA can be applied to fMRI time series from a region of interest [26]. That said, the main advantage we see of this approach is that it provides principled population level inference on optimal feature space and dimension which could be particularly useful for data-rich neuroimaging techniques (like M/EEG).
